# Bacterial associates of seed-parasitic wasps (Hymenoptera: *Megastigmus*)

**DOI:** 10.1186/s12866-014-0224-4

**Published:** 2014-09-25

**Authors:** Amber R Paulson, Patrick von Aderkas, Steve J Perlman

**Affiliations:** Department of Biology, University of Victoria, PO Box 3020, Station CSC, Victoria, BC V8W 3 N5 Canada

**Keywords:** *Burkholderia*, Endophytophagy, Galls, Microbiome, *Ralstonia*, *Rickettsia*, Seed parasitism, Symbiosis, *Wolbachia*

## Abstract

**Background:**

The success of herbivorous insects has been shaped largely by their association with microbes. Seed parasitism is an insect feeding strategy involving intimate contact and manipulation of a plant host. Little is known about the microbial associates of seed-parasitic insects. We characterized the bacterial symbionts of *Megastigmus* (Hymenoptera: Torymidae), a lineage of seed-parasitic chalcid wasps, with the goal of identifying microbes that might play an important role in aiding development within seeds, including supplementing insect nutrition or manipulating host trees. We screened multiple populations of seven species for common facultative inherited symbionts. We also performed culture independent surveys of larvae, pupae, and adults of *M. spermotrophus* using 454 pyrosequencing. This major pest of Douglas-fir is the best-studied *Megastigmus*, and was previously shown to manipulate its tree host into redirecting resources towards unfertilized ovules. Douglas-fir ovules and the parasitoid *Eurytoma* sp. were also surveyed using pyrosequencing to help elucidate possible transmission mechanisms of the microbial associates of *M. spermotrophus*.

**Results:**

Three wasp species harboured *Rickettsia*; two of these also harboured *Wolbachia*. Males and females were infected at similar frequencies, suggesting that these bacteria do not distort sex ratios. The *M. spermotrophus* microbiome is dominated by five bacterial OTUs, including lineages commonly found in other insect microbiomes and in environmental samples. The bacterial community associated with *M. spermotrophus* remained constant throughout wasp development and was dominated by a single OTU – a strain of *Ralstonia*, in the Betaproteobacteria, comprising over 55% of all bacterial OTUs from *Megastigmus* samples. This strain was also present in unparasitized ovules.

**Conclusions:**

This is the first report of *Ralstonia* being an abundant and potentially important member of an insect microbiome, although other closely-related Betaproteobacteria, such as *Burkholderia*, are important insect symbionts. We speculate that *Ralstonia* might play a role in nutrient recycling, perhaps by redirecting nitrogen. The developing wasp larva feeds on megagametophyte tissue, which contains the seed storage reserves and is especially rich in nitrogen. Future studies using *Ralstonia*-specific markers will determine its distribution in other *Megastigmus* species, its mode of transmission, and its role in wasp nutrition.

**Electronic supplementary material:**

The online version of this article (doi:10.1186/s12866-014-0224-4) contains supplementary material, which is available to authorized users.

## Background

One of the major reasons that insects are the most diverse and abundant animals on Earth is due to their coevolution with plants and the myriad strategies they have evolved to successfully feed on them [1]. Only recently have we come to appreciate that microbial endosymbionts of phytophagous insects have played a important role in this success [2,3], for example by providing essential metabolites and vitamins [4-8], breaking down cell wall components, such as lignocellulose [9], recycling nitrogenous waste [10] and detoxifying plant secondary metabolites [11,12]. Maternally transmitted intracellular symbionts are extremely common in herbivorous insects [3]. Obligate nutritional symbionts are usually found within specialized host-derived organs called bacteriomes and they often exhibit co-speciation with their host lineages, indicative of an ancient association stabilized by strict vertical transmission from mother to offspring [13,14]. In addition, many insects harbour facultative heritable endosymbionts that are not necessary for the development and reproduction of the host [14]. These symbionts have evolved diverse strategies to persist in their hosts, including manipulating reproduction, for example by inducing parthenogenesis [15]. Other facultative symbionts increase host fitness under certain conditions, and it is in this regard that they are potentially important in mediating plant-insect interactions [3,16,17]. For example, facultative inherited symbionts of pea aphids have been implicated in facilitating the colonization of novel host plants [18,19].

Gut microbes also play critical roles in plant-insect interactions. Some herbivorous insects are associated with essential communities of microbes found within gut chambers (e.g. termite, cockroach) [20,21] or crypts (e.g. true bugs) [22]. Several posthatch transmission mechanisms have evolved to ensure transmission of gut associates from generation to generation, such as egg-smearing [23], coprophagy [24] and capsule-mediated transmission [25]. In addition, some true bugs acquire their gut microbes *de novo* every generation from the environment [26-28]. Gut bacteria can affect a herbivore’s host range. For example, when the symbiont capsule from a stinkbug pest of soybean*, Megacopta punctatissima*, is exchanged with a non-pest species, *M. cribraria*, there is an increase in fitness of this species on soybean and a decrease in fitness of the pest species on soybean [29]. This implies that the obligate symbiont dictates the pest status of the host. Since some of the major lineages of gut symbionts have only recently been discovered and characterized, we are still in early days in our understanding of how associated microbial communities are able to shape plant-insect interactions [16].

There are many examples of nutritional symbiosis among phytophagous hymenopterans. Xylophagous woodwasps and horntails rely on a symbiotic fungus for cellulose-digestion and/or nutrition during larval stages [30,31] and woodwasps have also been found to be associated with cellulose degrading bacteria [32]. Leaf-cutter ants have also formed a symbiotic relationship with fungi, in which the ants cultivate and consume a mutualistic fungus on a substrate of foraged leaf fragments [33]. The honeybee, *Apis mellifera,* is known to be associated with a distinct microbiota [34-39], that is thought to be important for both bee health and nutrition [35,38], including pollen coat digestion. Arboreal herbivorous ants that subsist mainly on a nutrient-poor diet of sugary plant exudates and hemipteran honeydew secretions harbour gut symbionts, which aid in nutrition. These symbiotic gut microbes include bacteria that are related to nitrogen-fixing root-nodule bacteria [40-42]. Carpenter ants in the genus *Camponotus* have an obligate endosymbiont, the gammaproteobacterium *Blochmannia*, which is found in host-derived bacteriomes [43]. Sequencing of the *Blochmannia* genome suggests that this symbiont provides its host with essential amino acids [44,45]. There is also evidence that *Blochmannia* plays a role in nitrogen recycling by encoding urease [46].

Many insects have independently evolved the ability to feed from within plant issues, for example, as seed-feeders, gallers, or leaf-miners. This feeding style permits the larval stage access to internal plant tissues with relatively high nutrient content and low defence response, and often involves complex physiological and morphological modifications of host plant tissue, including differentiation of additional tissues (gall formation), *in situ* up-regulation and synthesis of proteins and sugars, translocation of nutrients to the insect feeding site and the formation of green islands (photosynthetically active areas surrounding leaf-mining insects during leaf senescence) [47-50]. However, the mechanisms controlling these complex modifications are not well understood; it remains an open question whether symbiotic microbes might have a role in these systems. An interesting study recently implicated bacterial symbionts in insect endophytophagy. Feeding by leaf-mining *Phyllonorycter blancardella* caterpillars prevents leaf senescence, resulting in characteristic islands of green tissue. These green islands are associated with increased levels of plant hormones [47,48,51], including cytokinins similar to those used by bacteria to manipulate plant physiology [52-54]. When leaf-miners were treated with antibiotics, the green-island phenotype failed to appear, suggesting that bacterial symbionts of *P. blancardella* might be involved in manipulation of the plant [51,55].

Seed chalcid wasps of the genus *Megastigmus* (Hymenoptera: Torymidae) provide an interesting system to explore the role of microbes in nutrition and host manipulation of endophytophagous insects. The genus *Megastigmus* contains 134 described species, of which more than 72 are tree and shrub seed feeders; the remaining species are thought to be mainly parasitoids of gall insects [56,57]. Seed infesting species of *Megastigmus* undergo their development within the seeds of plants, obtaining nourishment from the developing embryo and storage reserves within the megagametophyte [58]. The best-studied species, *M. spermotrophus*, is a major pest of Douglas-fir (*Pseudotsuga menziesii*). This insect has the ability to manipulate the seed development of Douglas-fir for its own reproductive success [59,60]. First, *M. spermotrophus* can re-direct unfertilized ovules that normally abort to continue developing. Ovules do not redirect resources back to the mother plant, but instead feed the insect [59]. Second, the developing larva acts like a ‘surrogate’ embryo, causing the continued accumulation of storage reserves in the megagametophyte, which provides nourishment for the larva [60]. The re-direction of unfertilized ovule development by the presence of the parasite can be partially explained by changes in seed hormone levels, especially cytokinins [61]. It is suspected that all *Megastigmus* species infesting Pinaceae hosts can manipulate seed development [62].

Do *Megastigmus* wasps contain bacterial associates, and if so could they play an important role in the endophytophagous lifestyle of the host? In this paper, we used two approaches to characterize the microbial symbionts of *Megastigmus*, with the long-term goal of understanding their role in host nutrition and manipulation. Using symbiont-specific primers we screened a large sample of sexual *Megastigmus* species and two parasitoids of *M. spermotrophus* for common insect facultative heritable endosymbionts [63]. We also used 16S rRNA bacterial amplicon pyrosequencing to perform an unbiased and in-depth survey of the microbes associated with different developmental stages of *M. spermotrophus* (the best-studied *Megastigmus* species and an important pest of Douglas-fir), Douglas-fir ovules and the parasitoid *Eurytoma* sp. There have not been any studies on the microbial associates of *Megastigmus* except for a recent study that showed that thelytokous parthenogenesis in *Megastigmus* is caused by the reproductive parasite *Wolbachia* [64].

## Results

### Common heritable endosymbiont infections in *Megastigmus*

**Figure 1:**
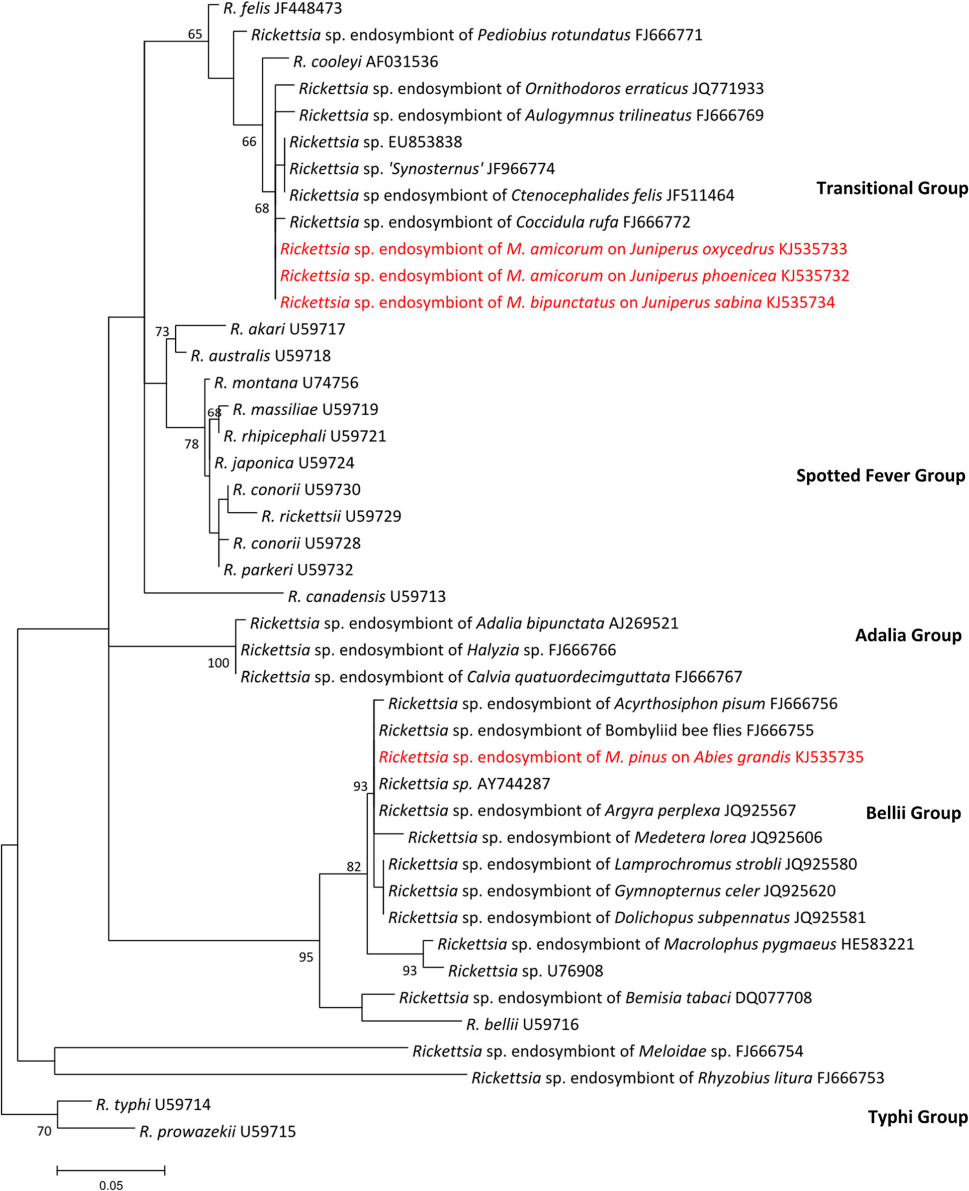
**Maximum likelihood phylogeny for** ***Rickettsia*** **citrate synthase sequence constructed using the Tamura 3-parameter plus gamma distributed rates among sites model of nucleotide substitution.** The sequences generated by this study are highlighted in red. Numbers next to the nodes indicate percentage of bootstrap support from 500 bootstrap replicates. Nodes without numbers received less than 65% bootstrap support.

**Figure 2:**
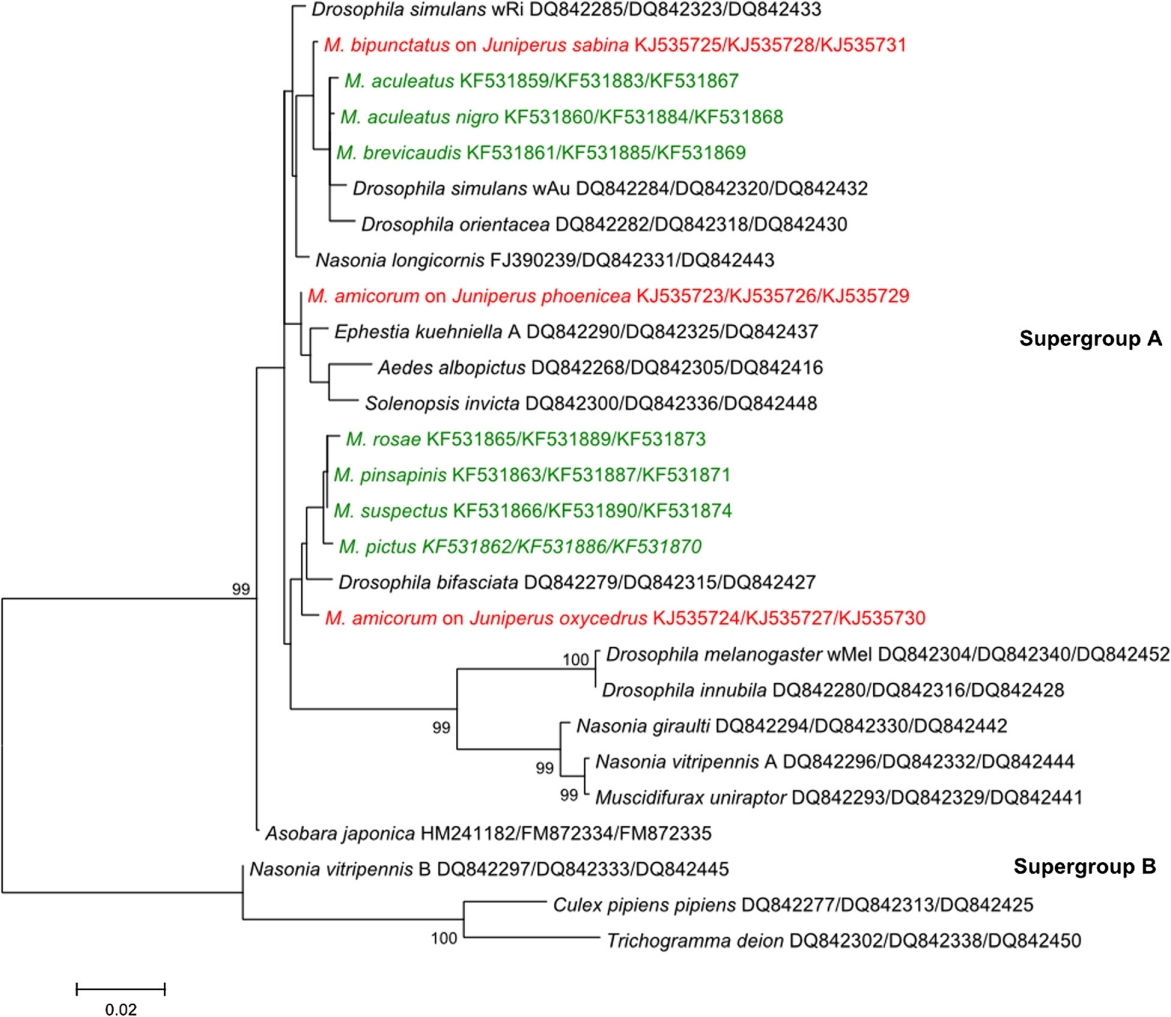
**Concatenated maximum likelihood phylogeny for** ***Wolbachia*** **coxA, ftsZ and gatB sequence constructed using the Tamura 3-parameter plus gamma distributed rates among sites model of nucleotide substitution.** Sequences generated by this study are red and sequences previously obtained from parthenogenetic *Megastigmus* are green [64]. Numbers next to the nodes indicate percentage of bootstrap support from 500 bootstrap replicates. Nodes without numbers received less than 65% bootstrap support.

Three species tested positive in our inherited symbiont screens, with infection frequencies ranging from 33–100% (Table 1). *Megastigmus milleri* harbours a strain of *Rickettsia* from the bellii clade (Figure 1) [GenBank:KJ353735]. *Megastigmus amicorum* and *M. bipunctatus* harbour a strain of *Rickettsia* that is allied with *R. felis,* i.e. in the ‘transitional’ group [65]. *Rickettsia* citrate synthase sequences from these two hosts were identical [GenBank:KJ353732 - KJ353734]. These two hosts also harboured supergroup A *Wolbachia* infections (Figure 2) [GenBank:KJ353723 - KJ353731]. *M. amicorum* collected from different host plants and locations (*Juniperus oxycedrus* from Corsica and *J. phoenicea* from mainland France) were 2% divergent in mitochondrial COI [GenBank:KJ535736 - KJ535737] and infected with different *Wolbachia* strains. There was no significant difference in the frequency of infection in males and females, nor did we find an association between *Wolbachia* and *Rickettsia* in coinfected species (Fisher’s exact tests, data not shown). *Arsenophonus*, *Cardinium*, and *Spiroplasma* were not detected in *Megastigmus* samples screened using PCR with symbiont-specific primers.

**Table 1:** ***Megastigmus*** **spp. and parasitoids screened for common heritable symbionts using PCR**

**Species**	**Host plant**	**Year**	**Location**	**N**	**Sample type**	***Wolbachia*** **positive**	***Rickettsia*** **positive**
Family: Pinaceae							
*M. schimitscheki*	*Cedrus atlantica*	2010	Petit Luberon, FR	15	Female		
*M. schimitscheki*	*Cedrus atlantica*	2009	Mont Ventoux, FR	14	Female		
*M. schimitscheki*	*Cedrus atlantica*	2010	Saou, FR	14	Female		
*M. schimitscheki*	*Cedrus atlantica*	2010	Gap, FR	15	Female		
*M. schimitscheki*	*Cedrus atlantica*	2008	Barjac, FR	15	Female		
*M. schimitscheki*	*Cedrus libani*	2005	Turkey	9	Female		
*M. rafni*	*Abies alba*	2009	Lespinassière, FR	15	Female		
*M. rafni*	*Abies alba*	2009	Pardailhan, FR	15	Female		
*M. rafni*	*Abies alba*	2010	Ventouret, FR	15	Female		
*M. rafni*	*Abies alba*	2004	Doubs, FR	9	Female		
*M. rafni*	*Abies nordmanniana*	2000	Rold Skov, DK	9	Female		
*M. rafni*	*Abies grandis*	2012	Vancouver Island, CAN	16	Female		
*M. rafni*	*Abies grandis*	2012	Vancouver Island, CAN	10	Male		
*M. milleri*	*Abies grandis*	2012	Vancouver Island, CAN	16	Female		75% (12)
*M. milleri*	*Abies grandis*	2012	Vancouver Island, CAN	10	Male		90% (9)
*M. spermotrophus*	*Pseudotsuga menziesii*	2011	British Columbia, CAN	26	Female		
*M. spermotrophus*	*Pseudotsuga menziesii*	2011	British Columbia, CAN	10	Larvae		
Family: Cupressaceae							
*M. watchli*	*Cupressus sempervirens*	2011	Sallèles du Bosc, FR	15	Female		
*M. watchli*	*Cupressus sempervirens*	2011	Monfavet, FR	15	Female		
*M. watchli*	*Cupressus sempervirens*	2011	Ruscas, FR	16	Female		
*M. watchli*	*Cupressus sempervirens*	1997	Aghois Ioannis, GR	10	Female		
*M. bipuncatatus*	*Juniperus sabina*	2011	Briançon, FR	10	Female	90% (9)	100% (10)
*M. bipuncatatus*	*Juniperus sabina*	2011	Pallon, FR	13	Female	38% (5)	54% (7)
*M. bipuncatatus*	*Juniperus sabina*	2011	Pallon, FR	10	Male	50% (5)	60% (6)
*M. amicorum*	*Juniperus phoenicea*	2011	Petit Luberon, FR	8	Female	100% (8)	100% (8)
*M. amicorum*	*Juniperus phoenicea*	2011	Luberon, FR	15	Female	100% (15)	93% (14)
*M. amicorum*	*Juniperus phoenicea*	2011	Luberon, FR	10	Male	80% (8)	70% (7)
*M. amicorum*	*Juniperus oxycedrus*	2009	Corsica, FR	10	Female	70% (7)	80% (8)
*M. amicorum*	*Juniperus oxycedrus*	2011	Corsica, FR	10	Female	80% (8)	100% (10)
*M. amicorum*	*Juniperus oxycedrus*	2011	Corsica, FR	9	Male	33% (3)	56% (5)
Parasitoids of *M. spermotrophus*							
*Eurytoma sp.*	-	2011	British Columbia, CAN	7	-		
*Mesopolobus sp.*	-	2011	British Columbia, CAN	16	-		

These species did not host *Arsenophonus, Cardinium*, or *Spiroplasma. Spiroplasma* was identified from *Eurytoma* sp. using 16S rRNA pyrosequencing.

### Microbial associates of *M. spermotrophus*

16S rRNA bacterial amplicon pyrosequencing of *M. spermotrophus* (adult females, larvae and pupae), adult *Eurytoma* sp. and *P. menziesii* ovules generated 81,207 raw reads with an average length of 422 bp (see Additional file 1) [BioProject: PRJNA239784]. Quality and chimera filtering removed approximately 27% of the reads. The assignment of operational taxonomic units (OTUs) resulted in 352 unique bacterial clusters after the removal of singletons. A total of 160 OTUs were assigned to the genus level. The average sequencing depth was 3,616 sequences per sample (minimum and maximum of 1,962 and 6,130 sequences per sample). Rarefaction analysis showed that for most of the *M. spermotrophus* samples the number of observed OTUs no longer exponentially increased after an approximate sampling depth of 3,000 sequences (see Additional file 2) and the average number of observed species was 60 ± 13 and the average Chao1 species diversity estimate was 71 ± 25.

Fifteen major OTUs form the core bacterial microbiome of *M. spermotrophus,* i.e. having a total relative abundance of 0.5% or greater (Table 2). These OTUs are from five bacterial classes: Betaproteobacteria, Gammaproteobacteria, Actinobacteria, Firmicutes and Alphaproteobacteria. Over 60% of the sequences from the *M. spermotrophus* samples were assigned to the genus *Ralstonia* spp. (61.57%). Other major OTUs were assigned to the genera *Acinetobacter* and *Corynebacterium* representing 17.20% and 4.44% of total relative abundance, respectively. Further investigation using BLAST searches against the Ribosomal Database Project (http://rdp.cme.msu.edu/) and GenBank’s 16S ribosomal RNA sequence database revealed that all but one of the major OTUs not assigned to the genus level were actually *Acinetobacter*, *Corynebacterium*, or *Ralstonia*. The unknown Firmicutes is most closely related to *Turicibacter*, a strictly anaerobic gram-positive bacteria in the family Erysipelotrichaceae [66]; this OTU represents 0.74% of the total relative abundance of the 16S rRNA sequences in the *M. spermotrophus* samples.

**Table 2:** **Major bacterial OTUs associated with** ***M. spermotrophus*** **(greater than 0.5% average relative abundance) based on 16S rRNA amplicons from pyrosequencing**

**Phylum**	**Class**	**Order**	**Family**	**Genus**	**Percent total relative abundance**
Proteobacteria	Betaproteobacteria	Burkholderiales	Oxalobacteraceae	*Ralstonia*	55.86
Proteobacteria	Gammaproteobacteria	Pseudomonadales	Moraxellaceae	*Acinetobacter*	16.28
Actinobacteria	Actinobacteria	Actinomycetales	Corynebacteriaceae	*Corynebacterium*	3.41
Proteobacteria	Betaproteobacteria	Burkholderiales	Oxalobacteraceae	*Ralstonia*	3.12
Proteobacteria	Betaproteobacteria	Burkholderiales	Oxalobacteraceae	*Ralstonia*	2.59
Proteobacteria					1.29
Actinobacteria	Actinobacteria	Actinomycetales	Corynebacteriaceae	*Corynebacterium*	1.03
Actinobacteria	Actinobacteria	Actinomycetales			0.95
Proteobacteria	Gammaproteobacteria	Pseudomonadales	Moraxellaceae	*Acinetobacter*	0.92
Firmicutes	Clostridia	Clostridiales	Clostridiaceae	*Anaerococcus*	0.79
Firmicutes					0.74
Proteobacteria					0.73
Proteobacteria	Betaproteobacteria				0.72
Firmicutes	Clostridia	Clostridiales	Clostridiaceae	*Anaerococcus*	0.52
Proteobacteria	Alphaproteobacteria	Rhizobiales	Bradyrhizobiaceae		0.50

**Figure 3:**
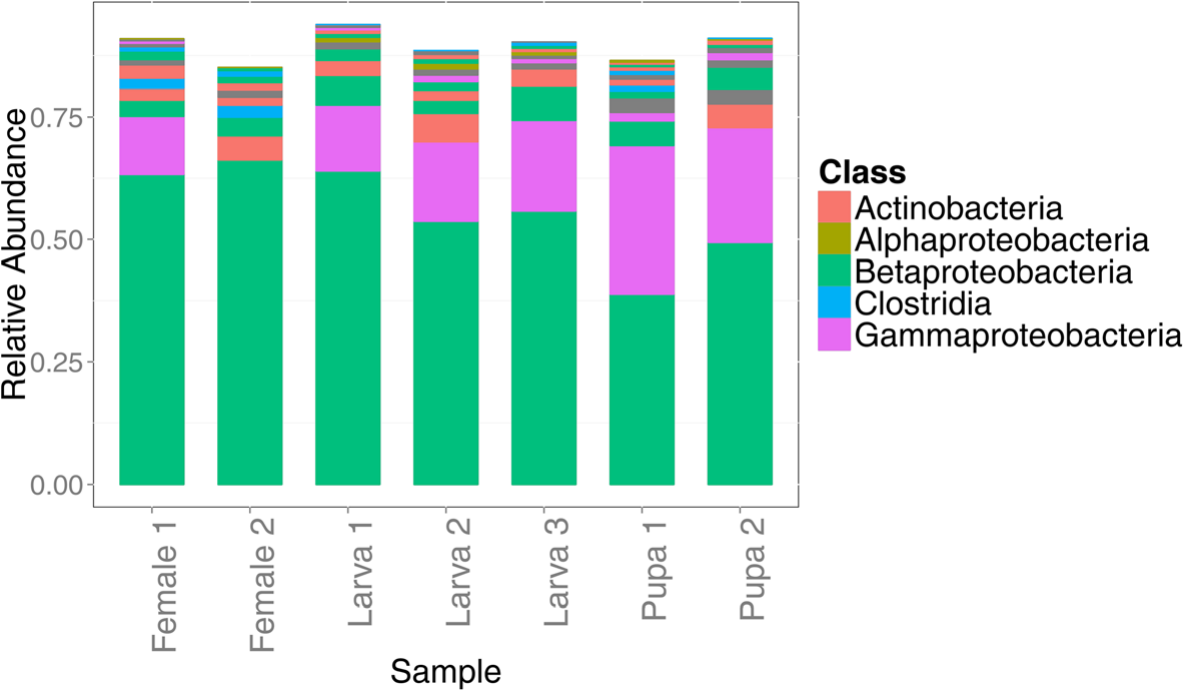
**Relative abundance of major bacterial OTUs associated with larvae, pupae and adult** ***M*** **.** ***spermotrophus*** **(total relative abundance greater than or equal to 0.5%) based on 16 rRNA sequence from pyrosequence.** Unknown classes are coloured grey.

The relative abundance of the major OTUs from the different developmental stages of *M. spermotrophus* was mostly conserved (Figure 3), and there was no difference in the core microbiomes of the different developmental stages, based on principle coordinate analysis of weighted or unweighted UniFrac phylogenetic distances (see Additional file 3). The total relative abundance of OTUs from the class Betaproteobacteria (all in the genus *Ralstonia*) ranged from 46.4 - 72.3%. One female sample contained only a very small proportion of OTUs assigned to the class Gammaproteobacteria (0.36% relative abundance) while the total relative abundance of Gammaproteobacteria ranged from 12.7 - 33.1% in the remaining samples. The total relative abundance of all OTUs within the class Actinobacteria (all in the genus *Corynebacterium*) ranged from 1.9 - 7.1%.

**Figure 4:**
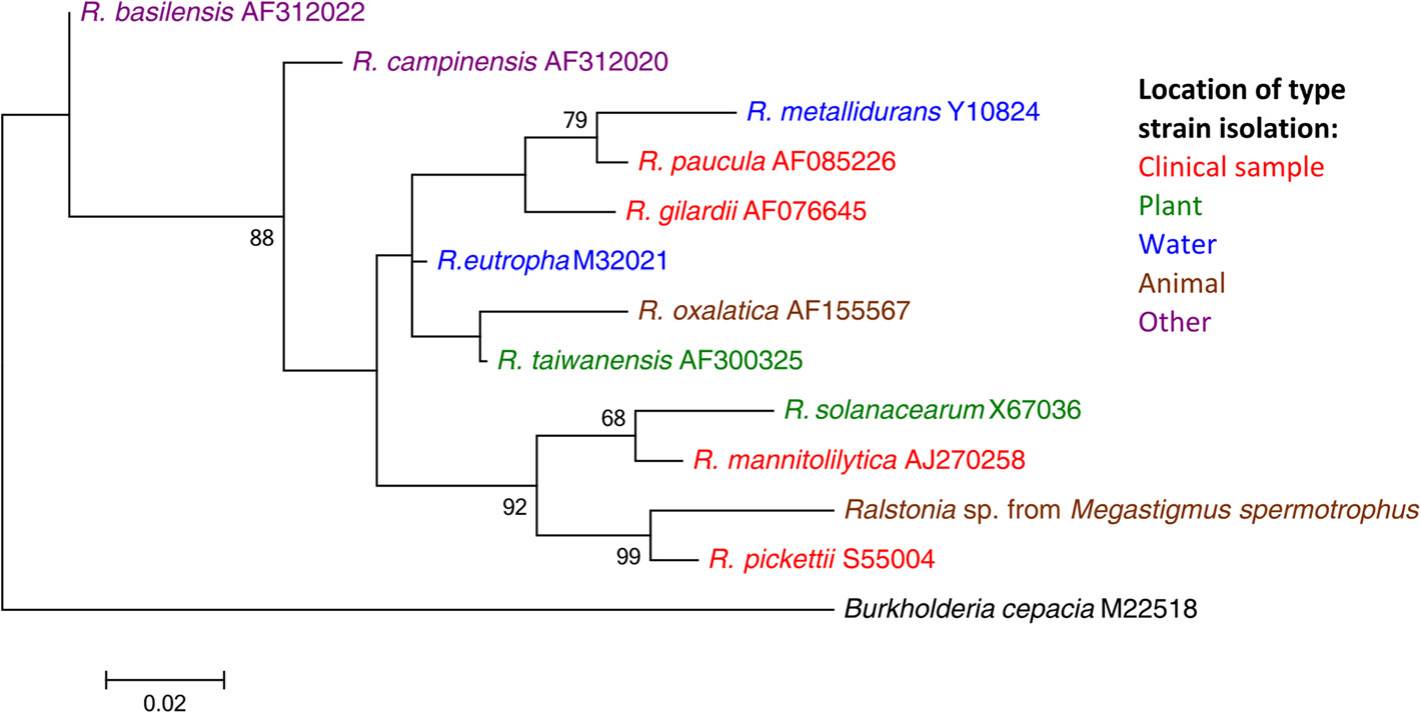
**Maximum likelihood phylogeny for** ***Ralstonia*** **16S rRNA sequence constructed using the Tamura-Nei with invariant sites and gamma distributed rate among sites model of nucleotide substitution.** Numbers next to the nodes indicate percentage of bootstrap support from 500 bootstrap replicates. Nodes without numbers received less than 65% bootstrap support.

A maximum likelihood phylogeny for *Ralstonia* was created using 16S rRNA sequence from the most abundant *Ralstonia* OTU in the pyrosequencing data set (Figure 4). Strong bootstrap support (0.99) clusters the *Ralstonia* isolated from *M. spermotrophus* with the human pathogen *R. pickettii* (sequence divergence = 3.3%).

Ovule samples were dominated by chloroplast rRNA (99.0%); the remaining OTUs included *Ralstonia* (0.8%) and *Acinetobacter* (0.2%). The *Eurytoma* parasitoid samples were dominated by one OTU, which is allied with inherited *Spiroplasma* in the *Ixodetis* group (see Additional file 4) [GenBank:KJ535740], (99.6%). The remaining OTUs were *Ralstonia*.

## Discussion

### Common heritable endosymbiont infections in *Megastigmus*

We found three sexual *Megastigmus* species infected with *Rickettsia*, and two of these same species infected with *Wolbachia*. None of the species was infected with *Arsenophonus*, *Spiroplasma*, or *Cardinium*. From this patchy distribution (i.e. high prevalence in some host populations and low prevalence or absence in others), we can likely conclude that none of these inherited symbionts are essential in host nutrition and/or manipulation.

It is not surprising that *Wolbachia* was detected, as it is the most common intracellular bacterial symbiont of insects [67]. *Wolbachia* are transmitted maternally, in the egg cytoplasm, and many strains have evolved strategies to increase the frequency of infected female hosts in the population. Reproductive manipulating strains of *Wolbachia* have been show to either cause cytoplasmic incompatibility or distort sex ratios by killing males or inducing parthenogenetic reproduction (i.e. clonal production of females) or feminization [68]. Parthenogenesis-inducing *Wolbachia* are common in Hymenoptera and have been characterized in several parasitoid [69] and cynipid gall wasps [70,71]. A recent study implicated *Wolbachia* in parthenogenetic reproduction in *Megastigmus*, with 10/10 asexual species infected [64]. Treating *M. pinsapinis* with the antibiotic tetracycline restored the production of males, strongly suggesting that *Wolbachia* is the causative agent of thelytoky in asexual *Megastigmus*. No sexual *Megastigmus* species were infected with *Wolbachia* in the Boivin et al. study [64]; however, we found infections in *M. amicorum* and *M. bipunctatus*. The *Wolbachia* strains that we identified from sexual *Megastigmus* are closely allied with those in asexual *Megastigmus*. It would be interesting to determine if parthenogenesis-induction in *Megastigmus* is due to the host or the particular *Wolbachia* strain.

*Rickettsia* infections were discovered in three species. Bacteria in the genus *Rickettsia* are well known for being insect-vectored vertebrate pathogens, such as the causal agents of Rocky Mountain spotted fever (*R. rickettsiae*) and typhus (*R. typhi*). However, recent surveys have uncovered many *Rickettsia* that are vertically transmitted symbionts of diverse arthropods, most of which do not feed on vertebrates [72]. Some *Rickettsia* symbionts have been shown to distort host sex ratios via male-killing [73] or parthenogenesis-induction [74]. The presence of *Rickettsia* and *Wolbachia* in males likely rules out sex ratio distortion in our study. Alternatively, facultative symbionts may benefit their hosts under some circumstances. For example, some *Wolbachia* and *Rickettsia* increase host fitness by providing protection against natural enemies [75,76].

Phylogenetic analysis shows that closely related *Rickettsia* and *Wolbachia* infect distantly related *Megastigmus* (Figures 1 and 2). This provides strong evidence of horizontal transmission over evolutionary timescales, and is a common pattern in facultative inherited symbionts of insects [14]. In most cases, it is not known how inherited symbionts colonize novel hosts; shared hosts and shared natural enemies have both been implicated [77-80]. Interestingly, for some inherited symbionts, horizontal transmission over ecological timescales may be quite common [19,81]. It would be useful to sequence more rapidly evolving *Rickettsia* genes, to determine if there was very recent transmission between *M. amicorum* and *M. bipunctatus*. Since both these species develop in junipers, we could speculate that horizontal transmission occurs via shared host plants; the Boivin et al. study of *Wolbachia* in asexual *Megastigmus* also found evidence for such host-plant-mediated transmission [64]. Plant-mediated transmission may be an important and underappreciated way for symbionts to colonize hosts. Indeed, a recent study showed that an inherited *Rickettsia* in the sweet potato whitefly can be transmitted via phloem [61]. Two strains of *Arsenophonus* that infect planthoppers are transmitted both transovarially and via plants, and both have been implicated in plant disease [82,83]. However, as far as we are aware, *interspecific* transmission via plants has not yet been demonstrated in any inherited symbionts.

### Microbial associates of *M. spermotrophus*

Our estimate of *M. spermotrophus* microbial species richness (60 ± 13 OTUs) fell within the range of other studies of insect microbiomes. Pollenivorous and predacious Hymenoptera (bees and wasps) harbour distinct bacterial communities with the lowest level of species richness (11.0 ± 5.4 OTUs/sample), while termites harbour the highest species diversity (89.5 ± 61.2 OTUs/sample), based on a recent meta-analysis [84]. A recent study estimated the diversity of bacteria associated with parasitoid wasps from the genus *Nasonia* ranged from 14 to 38 bacterial OTUs [85]. Pyrosequencing has been show to detect a greater number of OTUs compared to traditional methods, such as 16S rRNA clone sequencing [86]. This might explain why the estimated bacterial diversity associated with *M. spermotrophus* is comparably high because the *Nasonia* study and many previous insect microbiome surveys were done using 16S rRNA clone sequencing.

Despite a relatively high overall richness, only fifteen major OTUs are present with a total relative abundance of 0.5% or greater. The core bacterial community of *M. spermotrophus* can thus be considered to have a somewhat low diversity, characterized by bacterial OTUs that are commonly found associated with insect guts. The major OTUs associated with *M. spermotrophus* can be grouped into five distinct phylotypes: Betaproteobacteria (mostly *Ralstonia*), Gammaproteobacteria (mostly *Acinetobacter*), Actinobacteria (*Corynebacterium*), Firmicutes (mostly *Anaerococcus*) and Alphaproteobacteria (family Bradyrhizobiales). Most of these OTUs are related to bacteria that have been previously reported in insect guts, with *Acinetobacter* and *Corynebacterium* especially common (e.g. [85,87]). All of the major OTUs identified below the order level are bacteria that commonly occur in the environment, such as in soil [88] and in the rhizospere [89]. Similar results are commonly found with microbial associates of insects. For example, the microbial symbionts of *Tetraponera* ants are closely related to nitrogen-fixing root nodule bacteria [40]. The giant mesquite bug, *Thasus neocalifornicus* acquires an important mutualistic gut symbiont *de novo* every generation from the soil [27]. The presence of the same major OTUs in *M. spermotrophus* in ovule and even *Eurytoma* samples provides clues to the distribution and transmission of the *Megastigmus* microbiome; it suggests that it is derived from the environment, which, for the developing wasp, is the ovule. *Acinetobacter* and *Corynebacterium* have been previously cultured from within surface-sterilized seeds and ovules [90-92].

The *M. spermotrophus* microbiome appears to be highly conserved across development, as demonstrated by the UniFrac analysis, with all of the samples tightly grouped. This contrasts with a recent survey of microbial associates of three *Nasonia* species that found that bacterial species richness increased with development [85]. Like most higher Hymenoptera, the larvae of *M. spermotrophus* have a blind digestive system with the midgut and hind gut only uniting during the last larval instar. Prior to pupation all of the built-up wastes are voided in a fecal pellet, termed the meconium [93]. During metamorphosis the larval midgut epithelium is discarded and replaced by a new pupal epithelium [94]. If these bacteria are associated with the gut, how *M. spermotrophus* maintains its major associates throughout development is not known. Some insects, like true bugs, termites and cockroaches, have crypts or paunches associated with the gut that are thought to enhance persistence of the microbiota [6]. This physiological feature is not well characterized in the Hymenoptera, with the exception of some ants [95].

A single OTU assigned to the genus *Ralstonia* comprised over 55% of all sequences from the *M. spermotrophus* samples. The high abundance and persistence of *Ralstonia* throughout host development is a strong indicator that this bacterium is an important associate of *M. spermotrophus. Ralstonia* was also found to be associated with Douglas-fir ovules and the parasitoid *Eurytoma.* The genus *Ralstonia* contains species from ecological diverse niches, such as the plant pathogen *R. solanacearum*, the opportunistic human pathogen *R. pickettii* and the environmental isolate *R. eurytropha* [96]. A maximum likelihood phylogeny placed *M. spermotrophus* associated *Ralstonia* in a cluster with the human pathogen *R. pickettii* (Figure 4). To our knowledge, this is the first report of *Ralstonia* being a very abundant and potentially important component of an insect microbiome, although *Ralstonia* spp. have been previously reported from microbial surveys of insects, including the cotton bollworm (not published; accession # EU124821), *Bartonella*-positive fleas [97], an omnivorous carabid beetle [98] and the Potato Psyllid (as well as the faucet water used to water the potato plants) [99]. Recently, Husnik et al. also report the horizontal transfer of one *Ralstonia* gene into the genome of the mealybug *Planococcus citri* [100]. Also, *R. oxalatica* was isolated from the alimentary canal of an Indian earthworm [101].

A recent meta-analysis of 16S clone-library studies of insect associated microbes found that Betaproteobacteria contributed over 50% to all sequences from Hymenoptera [84]. The most common bacterial phylotype identified from solitary bee species, was a Betaproteobacteria from the genus *Burkholderia* [35], which is closely related to *Ralstonia. Burkholderia* spp. have also been identified as important mutualists of some phytophagous true bugs (suborder Heteroptera), where they reside in gut crypts [26-28,102,103].

The developing *M. spermotrophus* larva feeds on megagametophyte tissue, which contains all of the seed storage reserves, primarily in the form of starch, triacylglycerols, and nitrogen rich proteins [104,105]. Therefore, *Ralstonia* and other microbial associates of *M. spermotrophus* would not likely play a role in supplementing this already rich diet with missing essential nutrients but instead may play a role in nutrient recycling. Parasitism by *M. spermotrophus* results in the formation of a nutrient sink, in which the larva and associated microbes are nourished by storage reserves of the megagametophyte. The reserves are intended to provide nourishment for the developing seedling or to be re-absorbed by the mother plant in the event of megagametophyte abortion. In loblolly pine, more than half of the nitrogen in megagametophytes comes from the amino acid arginine [106]. Insects use the enzyme arginase to hydrolyze arginine into ornithine and urea [107]. Excretion of urea would result in the substantial loss of nitrogen, especially since larvae must undergo extended periods of diapause. Very few insects are known to produce urease, the enzyme required to convert urea into ammonium for subsequent amino acid biosynthesis [108]. We speculate that *Ralstonia* or other microbial associates of *M. spermotrophus* might play an important role in nitrogen recycling by producing urease or other key enzymes missing from the host genome. Many insect symbionts have been suggested to promote increased availability of nitrogen in a variety of ways [5]. For example, *Blochmannia* and *Blattabacterium*, the obligate nutritional symbionts of carpenter ants and cockroaches, respectively, use ureases to recycle nitrogen from urea [109,110]. Nitrogen recycling by symbionts has also been shown to be important during diapause in the shield bug, *Parastrachia japonensi* [111].

It is also tempting to speculate that *Ralstonia* could potentially play a role in plant manipulation. Another *Ralstonia* species, *R. taiwanensi*, has been shown to be capable of nodulating and fixing nitrogen in *Mimosa* spp. [112], which implies an ability to manipulate plant physiology. Alternatively, *Ralstonia* may not be a key associate of *Megastigmus* species in general, but rather a microbe that is found in the seed environment that encodes enzymes required for the catabolism of seed storage molecules or other essential pathways required for the seed feeding lifestyle of *M. spermotrophus*.

Now that *Ralstonia* has been identified as a likely symbiont of *M. spermotrophus*, further targeted surveys using *Ralstonia*-specific PCR primers would be helpful in determining its prevalence in other populations of *M. spermotrophus*, in other *Megastigmus* species, and in associated plants. The development of strain-specific markers for fluorescence *in situ* hybridization would also be useful for localizing *Ralstonia* on or within *M. spermotrophus* and the ovule, and following its transmission throughout its life cycle*.* It would also be interesting to examine *Ralstonia*’s role in nitrogen recycling, for example by identifying and following the expression of ureases and other key enzymes during *M. spermotrophus* development.

## Conclusions

In this study two different approaches were used to survey *Megastigmus* for microbial symbionts. The directed PCR screens identified the presence of two common heritable symbionts, *Wolbachia* and *Rickettsia*; these are not likely distorting sex ratios in the sexual *Megastigmus* species surveyed in this study. Pyrosequencing was used to characterize the core microbiome of the Douglas-fir seed chalcid, *M. spermotrophus*, which is dominated by *Ralstonia*, a microbe that has not been previosly characterized as an important microbial associated of an insect. Interestingly, *Ralstonia* was also present in ovule and *Eurytoma* samples, indicating its prevalence within the niche of the ovule and potential horizontal transmission route from host to parasitoid.

This initial characterization of microbial associates of *Megastigmus* did not provide any insight into the potential involvement in host manipulation, although the maintenance of a consistent microbiome from larvae to adult suggests that microbes may be vital to the development and reproduction of *M. spermotrophus*. Many new questions are inspired by these findings, such as, how is the microbiome of *M. spermotrophus* maintained and transmitted? How widespread is the association with *Ralstonia*? What is the effect of heritable symbionts in sexual *Megastigmus*?

## Methods

### Insect samples

Several species of *Megastigmus* and their parasitoids were screened for common heritable symbionts using PCR. Adult insects were reared from seeds that were collected from forest stands in France, Greece, Denmark and Turkey from 1997 to 2011; detailed information on sample species is listed in Table 1. Also, larvae of *M. spermotrophus* were dissected from infested seed collected in 2011 from seed orchards located throughout British Columbia. Adult *M. spermotrophus* were reared from this same seed. Any *Eurytoma* sp. parasitoids that emerged were also collected. Wild adult female *M. spermotrophus* were collected from trees located on the University of Victoria campus in Victoria, BC. Whole insect samples were stored in 95% ethanol at −20°C until DNA extraction.

For 16S rRNA bacterial amplicon pyrosequencing, *M. spermotrophus* and their parasitoids were obtained in 2011 from heavily infested seed from the Mt. Newton Seed Orchard, located in Saanichton, BC. The seeds were placed at room temperature to hasten the development of larvae and adult emergence. Larvae as well as approximately one-week-old pupae were extracted from surface-sterilized seeds. Adult female *M. spermotrophus* and adult *Eurytoma* sp. were collected upon emergence about two and three weeks later, respectively. Samples of uninfested ovules were also collected from surface-sterilized seeds.

### DNA extraction

Whole insects were rinsed several times with sterile water and allowed to air dry. The samples were then placed individually into 2 mL Micro tubes (Sarstedt) with 100 μL of PrepMan Ultra Reagent (Applied Biosystems, USA) and approximately twenty 1.0 mm diameter zirconia or silica beads (BioSpec Products). Samples were homogenized using the Mini-Beadbeater-16 (BioSpec Products) on maximum (3450 oscillations/min) for two 20–30 second cycles separated by 30 seconds of centrifugation at 13,000 × g. The samples were then incubated at 100°C for ten minutes, then cooled to room temperature for one minute, then centrifuged for three minutes at 13,000 × g and transferred into new Eppendorf tubes. DNA samples used for pyrosequencing were purified by precipitation in cold isopropanol and then washed with 70% ethanol and re-suspended in TE buffer (pH = 7.5). A NanoDrop 2000 Spectrophotometer (Thermo Scientific) was used to determine the DNA concentration and quality. The quality of the DNA extract was also checked by successful PCR amplification of the mitochondrial cytochrome oxidase subunit I (COI) gene using standard primers for invertebrates (see Additional file 5). All DNA extracts were stored at −20°C.

### Directed PCR

Directed PCRs were conducted using either Invitrogen or ABM PCR Taq and reagents. Symbiont-specific primer-pairs were used to screen the samples for the presence of common heritable symbionts (see Additional file 5) with the following infected insects used as positive controls: *Drosophila neotestacea* (*Wolbachia* and *Spiroplasma* positive), *Macrosteles quadrilineatus* (*Arsenophonus* and *Cardinium* positive), and *Ctenocephalides felis* (*Rickettsia* positive). Sterile water was used as a negative control. Positive PCR products were separated on 1% agarose gel, stained with eithidium bromide and visualized under UV light.

Five microlitres of DNA from each individual extraction within a sample subset (individuals of the same species, sample type, location and year) were pooled (total of 32 pooled samples) and then screened using each primer set. If a positive PCR product was amplified from a pooled sample then each individual sample was screened for presence or absence of the corresponding symbiont using the same primer set. Positive PCR products were validated by sequencing representative amplicons in both directions. Purification and sequencing of PCR products were completed at Macrogen USA (Maryland). Forward and reverse sequences were aligned using MUSCLE and manually edited using the software Geneious (v6.1.3) (Biomatters) to create high-quality consensus sequences. A portion of mitochondrial COI was sequenced from one representative female of every symbiont-positive population, using *Megastigmus*-specific primers (see Additional file 5) and compared with other *Megastigmus* sequences deposited in GenBank. Percent divergence between COI sequences from *M. amicorum* populations was calculated using MEGA 5.1 [113].

### Phylogenetic analysis of *Rickettsia* and *Wolbachia* infecting *Megastigmus*

A number of additional symbiont genes were amplified via PCR and sequenced: citrate synthase gene (gltA) for *Rickettsia*, and coxA, and gatB for *Wolbachia* (see Additional file 5). Phylogenies were re-constructed using sequences generated in this study and a sample of sequences obtained from GenBank. For *Wolbachia*, a sample of sequences obtained from an independent study of *Wolbachia* in parthenogenetic *Megastigmus* was also included [64]. Sequences were aligned using ClustalW, visually inspected and trimmed when necessary. A maximum-likelihood tree was generated using the Tamura 3-parameter model plus gamma distributed rates among sites (best substitution model identified by MEGA), with MEGA 5.1 [113], bootstrapped 500 times.

### Bacterial tag-encoded FLX amplicon pyrosequencing

Three replicates of five sample types were submitted for bacterial tag-encoded FLX 454-pyrosequencing (bTEFAP): *M. spermotrophus* larvae, pupae and adult females, *Eurytoma* sp. adults and *P. menziesii* ovules. Although the 27 F/519R primer set is not ideal for characterizing bacterial 16S rRNA sequence from plant tissue due to chloroplast DNA contamination [114,115], we included ovule samples in order to see if any trace endophytic bacteria could be found after post-sequencing removal of plastid sequences. Inhibitor removal and bTEFAP were completed by MR. DNA Laboratories (Shallowater, TX). Inhibitor removal involved the use of the PowerClean DNA Clean-up kit (MO BIO Laboratories, Inc., Carlsbad, CA) according to the manufacturer’s protocol. The methods used for bTEFAP are previously described in Palavesam et al. (2012) and Shange et al. (2012) [116,117] and were originally described by Dowd et al. (2008) [118]. Briefly, a single-step PCR was done using the following temperature profile: 94°C for 3 minutes, followed by 28 cycles of 94°C for 30 seconds, 53°C for 40 seconds and 72°C for 1 minute, with a final elongation step at 72°C for 5 minutes using HotStarTaq Plus Master Mix Kit (Qiagen, Valencia, CA). The 16S universal bacterial primers 27Fmod (5’-AGRGTTTGATCMTGGCTCAG-3’) and 519Rmodbio (5’-GTNTTACNGCGGCKGCTG-3’) were used to amplify a 500 bp region of the 16S rRNA gene spanning the V1-V3 regions. The PCR products from each of the different samples were mixed in equal concentrations and then purified using Agencourt Ampure beads (Agencourt Bioscience Corporation, MA, USA). Following the manufacturer’s guidelines, sequencing was conducted using the Roche 454 FLX titanium platform (Roche, Indianapolis, IN).

### Qiime pipeline

The 454 generated Standard Format Flowgram (SFF) file was converted into a SFF text file using Mothur (v1.23.0) [119]. The open source software package Quantitative Insights Into Microbial Ecology (QIIME v1.6.0) was used to process the sequence data [120]. The raw sequencing data was filtered using the following parameters: minimum sequence length of 100 bp, maximum sequence length of 2,000 bp and maximum homopolymer region of eight. Also, any sequences with an average quality score below 25 or any ambiguous bases were discarded. This filtering step reduced the number of total sequences from 81,207 to 60,543. The 454 data were then denoised to reduce the number of erroneous OTUs [121]. Chimera detection was done independently of QIIME by implementing UCHIME through the USEARCH (v6.0.307) program [122]. The sequences were compared against the Gold database (http://www.drive5.com/usearch/manual/otupipe.html, downloaded February 13, 2013). Chimeric sequences (1,190 or 1.97%) were gleaned from the data set.

OTUs were picked with the UCLUST method with the optimal option indicated. Similar sequences were clustered at the default level of 0.97 [123]. Taxonomy was assigned to representative sequences using the RDP Classifier 2.2 method at the 0.9 confidence level [124]. Taxonomies were based on the Greengenes database (ftp://greengenes.microbio.me/greengenes_release/gg_12_10/, downloaded February 1, 2013) [125,126].

Originally, the PyNast method was used to align the representative sequences to a pre-aligned database; however, this method resulted in poor overall alignment. Alternatively, representative sequences were aligned to a Stockholm format reference of pre-aligned sequences and secondary structures using Infernal [127]. The aligned sequences were filtered to remove common gap positions, with the gap filter threshold set to 0.8 and the entropy threshold set to 0.10. An approximately-maximum-likelihood phylogenetic tree was created using FastTree 2.1.3 [128]. An OTU table in Biom format was created and then split at the highest taxonomic ranking to remove unclassified OTUs (likely remnant chimeric sequences). Singletons were removed from the Biom table. Alpha diversity results were generated using a rarefaction depth of 5,000. In order to identify possible outliers (i.e., samples that contain unusual or unexpected OTUs), the microbiome data were visualized using a correspondence analysis biplot [129]. One pupal sample (P1) and one female sample (F4) were found to be associated with distinct OTUs that did not cluster with the remaining samples. Sample P1 had a relatively elevated species richness compared to the other samples, likely originating from environmental contamination (data not shown). Sample F4 contained bacteria typical of human contamination. Subsequently these two samples were removed from further analysis.

Data exploration, visualization and analyses were performed in R (v3.0.1) [130] on RStudio (v0.97.336) (www.rstudio.com, downloaded August 5, 2013), mainly using the *Phyloseq* R-package (v1.5.19) [131]. Data were rarefied to an equal sampling depth of 1,962 prior to community analysis. Initial correspondence analysis and biplots were generated using the *Ade4* R-package (v1.5-2) [132]. Principle component analysis was completed using unweighted and weighted UniFrac distances [133,134].

In order to obtain longer 16S rRNA fragments for phylogenetic analysis from the *Spiroplasma* strain infecting *Eurytoma*, general 16S rRNA amplicons were generated using the primers 63 F (5’-CAGGCCTAACACATGCAAGTC-3’) [135] and 907R (5’-CCGTCAATTCCTTTRAGTTT-3’) [136]. Amplicons were then cloned using the Strataclone kit with Solopack Competent cells (Stratagene). Transformation was validated with PCR using M13F (5’- CACGACGTTGTAAAACGAC-3’) and M13R (5’-GGATAACAATTTCACACAGG-3’). Eight clones were sent for sequencing and one representative *Spiroplasma* 16S rRNA sequence was used for further analysis. Attempts to clone longer *Ralstonia* 16S rRNA fragments were not successful.

*Ralstonia* sequence from the most abundant OTU in the pyrosequencing data was used to generate a 16S rRNA phylogeny, along with representative *Ralstonia* species and outgroup sequences, obtained from GenBank. Maximum likelihood analysis was performed as above, except using the Tamura-Nei model with invariant sites and gamma rate distribution among sites.

## Additional files

Additional file 1
**Summary of 454 16S rRNA sequence data.** Summary of sequence data from tag encoded FLX 454-pyrosequencing of 16S rRNA from *M. spermotrophus*, *Eurytoma* sp. and *P. menziesii* ovule samples. Additional file 2
**Observed species and Chao1 species diversity estimator rarefaction curves.** Observed species richness and Chao1 species diversity estimator rarefaction curves for bacteria associated with different life stages of *M. spermotrophus*, based on 16S rRNA pyrosequencing. Additional file 3
**Analysis of phylogenetic distances.** Analysis of phylogenetic distances (UniFrac) for all OTUs associated with different developmental stages of *M. spermotrophus* based on 16S rRNA amplicon pyrosequence. Additional file 4
**Maximum likelihood phylogeny for** ***Spiroplasma*** **16S rRNA.** Maximum likelihood phylogeny for *Spiroplasma* 16S rRNA sequence constructed using the general time reversible model of nucleotide substitution with gamma distributed rates among sites. The sequence generated in this study is highlighted in red. Numbers next to the nodes indicate percentage of bootstrap support from 500 bootstrap replicates. Nodes without numbers received less than 65% bootstrap support. Additional file 5
**List of PCR primers and reactions conditions.** List of PCR primers and reactions conditions used to generate COI sequence from *Megastigmus* spp. and screen for common heritable symbiont infections.
