# Supplementary material for: Bacterial associates of seed-parasitic wasps (Hymenoptera: Megastigmus)
Source: BMC Microbiol. 2014 Sep 25;14:224. doi: 10.1186/s12866-014-0224-4 (PMC4197294; doi:10.1186/s12866-014-0224-4)
Supplement: Additional file 1 — Summary of 454 16S rRNA sequence data. Summary of sequence data from tag encoded FLX 454-pyrosequencing of 16S rRNA from M. spermotrophus, Eurytoma sp. and P. menziesii ovule samples. [file 12866_2014_224_MOESM1_ESM.pdf]

Summary of 454 16S rRNA sequence data

454 Sequence summary	
Number of raw input reads:	81,207
Raw length min/max/avg:	100/633/422.3
Number of filtered reads:	60,543
Filtered length min/max/avg:	72/605/394.3
Number of chimeric sequences:	1,190
Number of singletons:	183
Number of unclassified sequences:	4,932
OTU Assignments	
Number of OTUs assigned to kingdom	352
Number of OTUS assigned to phylum	255
Number of OTUS assigned to class	248
Number of OTUS assigned to order	233
Number of OTUs assigned to family	217
Number of OTUs assigned to genus	160
Seqs/sample summary	
Number of Samples:	15
Min:	1,962
Max:	6,130
Median:	2,974
Mean:	3,616
Standard Deviation:	1,415
