# Supplementary figures and images for: Bacterial associates of seed-parasitic wasps (Hymenoptera: Megastigmus)
Source: BMC Microbiol. 2014 Sep 25;14:224. doi: 10.1186/s12866-014-0224-4 (PMC4197294; doi:10.1186/s12866-014-0224-4)

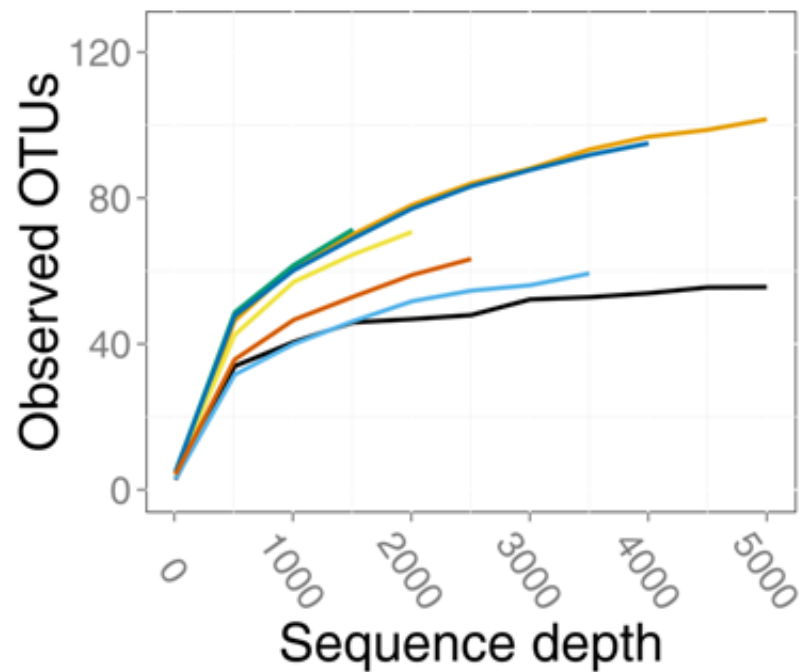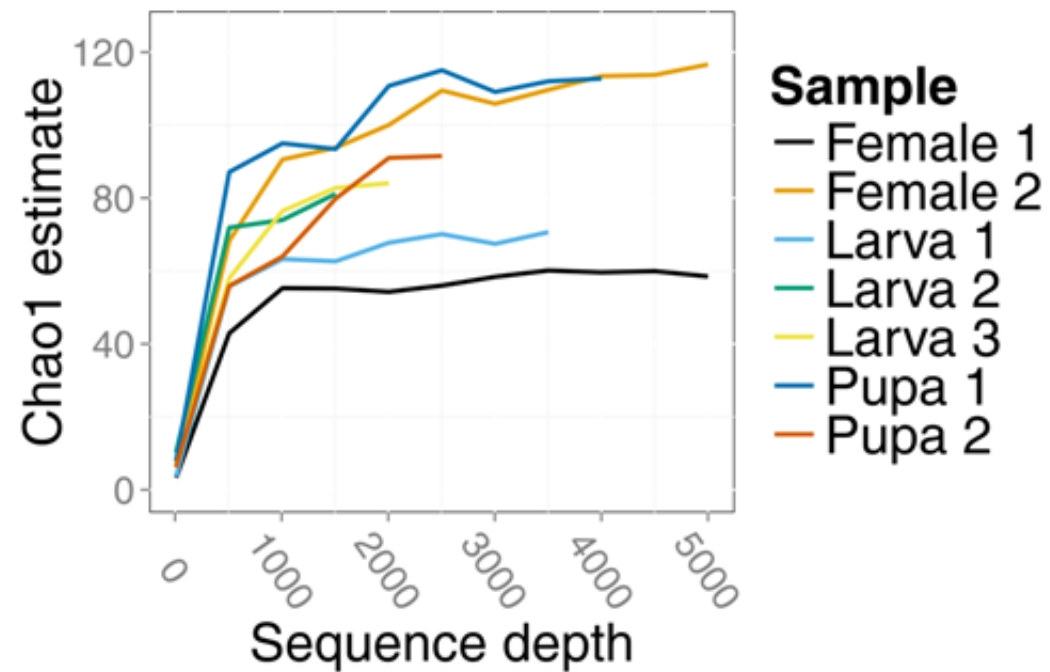

Supplement: Additional file 2 — Observed species and Chao1 species diversity estimator rarefaction curves. Observed species richness and Chao1 species diversity estimator rarefaction curves for bacteria associated with different life stages of M. spermotrophus, based on 16S rRNA pyrosequencing. [file 12866_2014_224_MOESM2_ESM.pdf]

# Unweighted UniFrac

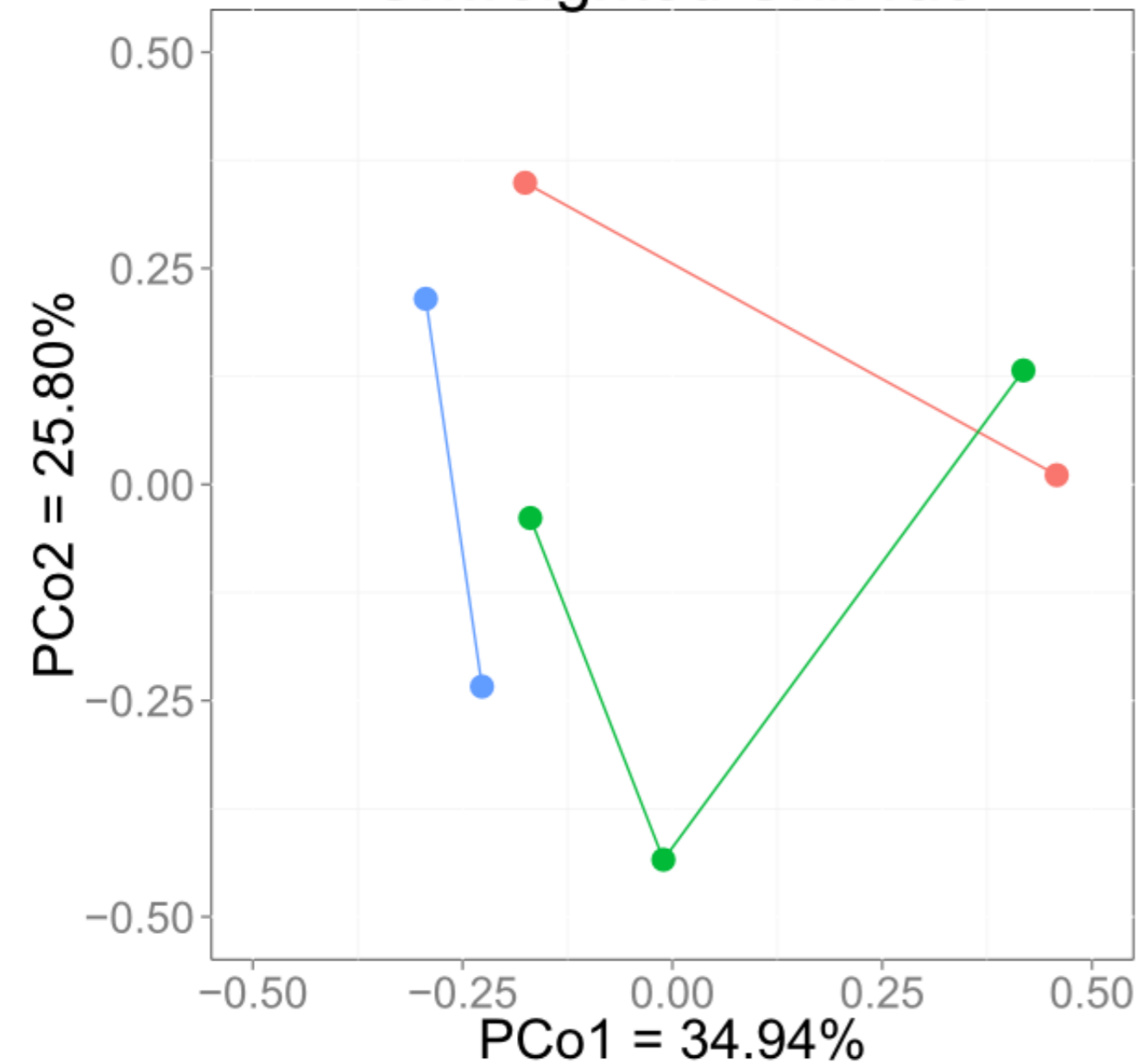

# Weighted UniFrac

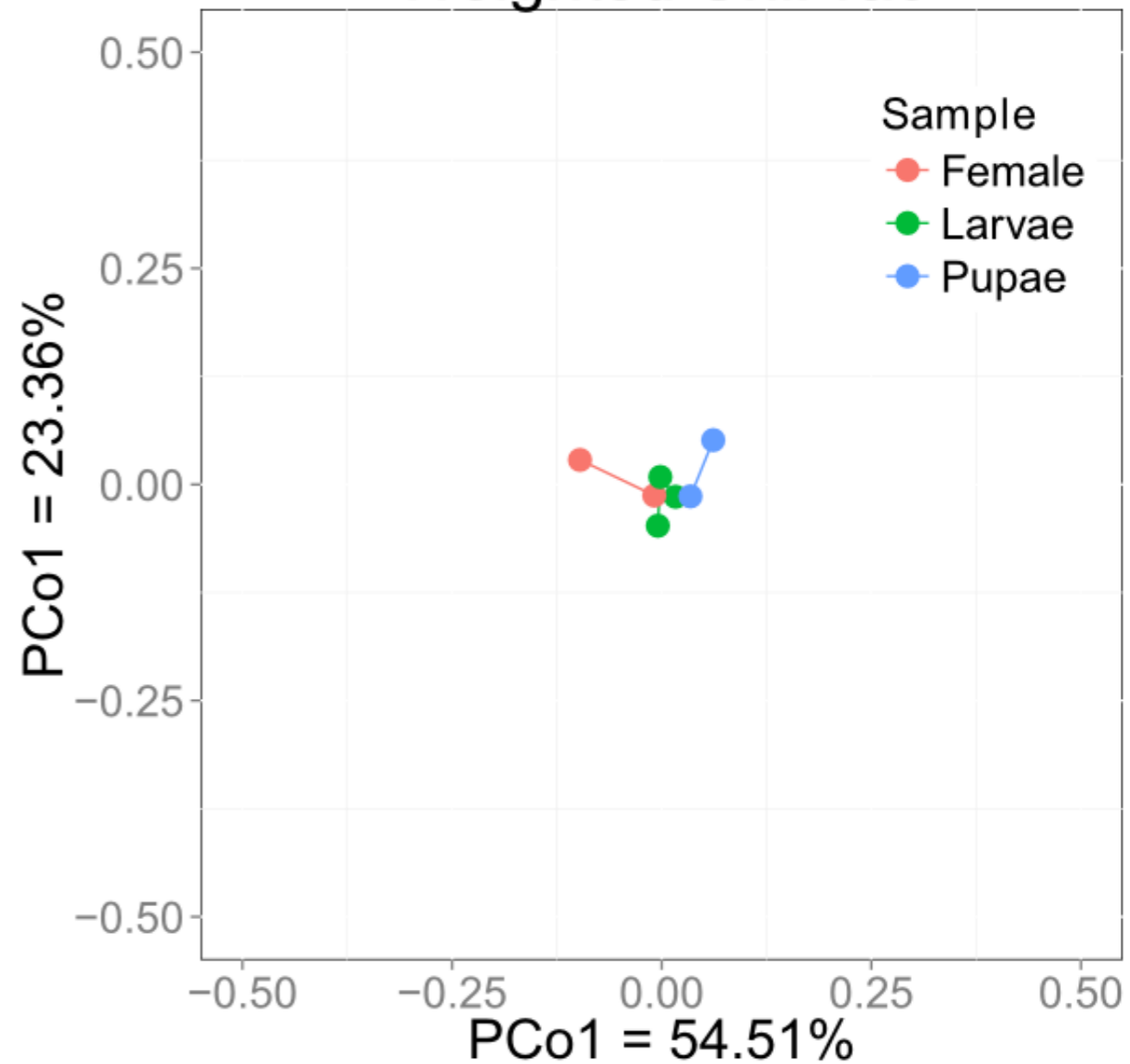

Supplement: Additional file 3 — Analysis of phylogenetic distances. Analysis of phylogenetic distances (UniFrac) for all OTUs associated with different developmental stages of M. spermotrophus based on 16S rRNA amplicon pyrosequence. [file 12866_2014_224_MOESM3_ESM.pdf]

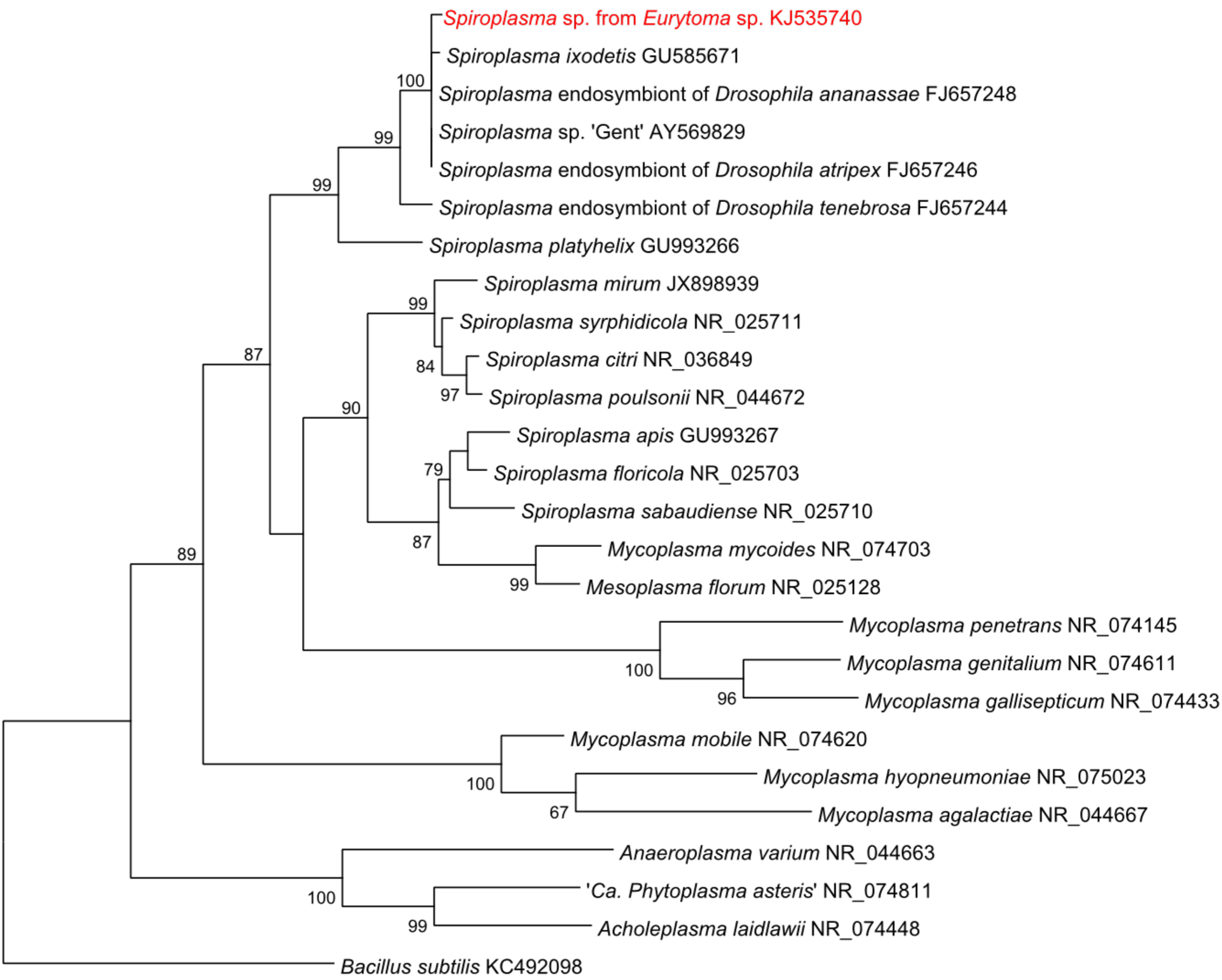

0.05

Supplement: Additional file 4 — Maximum likelihood phylogeny for Spiroplasma 16S rRNA. Maximum likelihood phylogeny for Spiroplasma 16S rRNA sequence constructed using the general time reversible model of nucleotide substitution with gamma distributed rates among sites. The sequence generated in this study is highlighted in red. Numbers next to the nodes indicate percentage of bootstrap support from 500 bootstrap replicates. Nodes without numbers received less than 65% bootstrap support. [file 12866_2014_224_MOESM4_ESM.pdf]
