# Supplementary material for: Bacterial associates of seed-parasitic wasps (Hymenoptera: Megastigmus)
Source: BMC Microbiol. 2014 Sep 25;14:224. doi: 10.1186/s12866-014-0224-4 (PMC4197294; doi:10.1186/s12866-014-0224-4)
Supplement: Additional file 5 — List of PCR primers and reactions conditions. List of PCR primers and reactions conditions used to generate COI sequence from Megastigmus spp. and screen for common heritable symbiont infections. [file 12866_2014_224_MOESM5_ESM.pdf]

List of PCR primers and reaction conditions

Target	Target gene	Primer name	Primer sequence (5'-3')	Product size (bp)	Temperature Profile	Refs.*
Invertebrates	COI	LCO1490<br>HCO	GGTCAACAAATCATAAAGATATTGG<br>TAAACTTCAGGGTGACCAAAAAATCA	600	95°C 3:00; 35x(94°C 1:00, 54°C 1:00, 72°C 1:30); 72°C 10:00	1
Megastigmus spp.	COI	Ana<br>Will	TCCAAAAATTGCAAATACAGC<br>TTCCTGATATAGCATTTCCTCG	900	95°C 3:00; 35x(94°C 1:00, 54°C 1:00, 72°C 1:30); 72°C 10:00	2
Arsenophonus	16S	ArSF<br>ArSR3	GGGTTGTAAAGTACTTTCAGTCGT<br>CCTYTATCTCTAAAGGMTTCGCTGGATG	580-800	95°C 2:00; 35x(94°C 0:30, 52°C 0:30, 72°C 1:30); 72°C 5:00	3
Cardinium	16S	CLOf<br>CLOr1	GCGGTGTAAAATGAGCGTG<br>ACCTMTTCTTAACTCAAGCCT	450	94°C 4:00; 35x(94°C 0:40, 57°C 0:40, 72°C 0:45); 72°C 5:00	4
Rickettsia	gltA	Rp877p<br>Rp1258n	GGGGACCTGCTCACGGCGG<br>ATTGCAAAAAGTACAGTGAACA	381	95°C 3:00; 35x(95°C 0:20, 48°C 0:30, 60°C 2:00); 72°C 10:00	5
Rickettsia	16S	RSSUf<br>RSSUr	GCTTTCAAAACTACTAATCTA<br>AAAAGCATCTCTGCGATCCG	380	95°C 3:00; 35x(95°C 0:60, 51°C 0:45, 72°C 1:00); 72°C 5:00	6
Spiroplasma *poulsonii, kunkelii, citri*	p58	p58-f<br>p58-r	GTTGGTTGAATAATATCTGTTG<br>GATGGTGCTAAATTATATTGAC	793	95°C 3:00; 35x(94°C 1:00, 54°C 1:00, 72°C 1:30); 72°C 10:00	7
Spiroplasma *ixodetis*	16S	SpixoF<br>SpixoR	TTAGGGGCTCAACCCCTAACC<br>TCTGGCATTGCCAACTCTC	810	95°C 2:00; 35X (94°C 0:30, 52°C 0:30, 72°C 1:30); 72°C 5:00	3
Wolbachia	coxA	coxA_F1<br>coxA_R1	TTGGRGCRATYAACTTTATAG<br>CTAAAGACTTTKACRCCAGT	402	94°C 2:00; 36x(94°C 0:30, 59°C 0:30, 72°C 1:30); 70°C 10:00	8
Wolbachia	ftsZ	ftsZ_F1<br>ftsZ_R1	ATYATGGARCATATAAARGATAG<br>TCRAGYAATGGATTRGATAT	1200	94°C 3:00; 35x(94°C 0:30, 54°C 0:45, 70°C 1:30); 70°C 10:00	8
Wolbachia	gatB	gatB_F1<br>gatB_R1	GAKTTAAAYCGYGCAGGBGTT<br>TGGYAAYTCRGGYAAAGATGA	369	94°C 2:00; 36x(94°C 0:30, 55°C 0:30, 72°C 1:30); 70°C 10:00	8

* 1. Folmer O, Black M, Hoeh W, Lut R, Vrijenhoek R: **DNA primers for amplification of mitochondrial cytochrome c oxidase subunit I from diverse metazoan invertebrates.** *Mol Mar Biol Biotechnol* 1994, **3**: 294-299.

2. Marie-Anne Auger-Rozenberg, unpublished.

3. Duron O, Bouchon D, Boutin S, Bellamy L, Zhou L, Engelstädter J, Hurst GD: **The diversity of reproductive parasites among arthropods: *Wolbachia* do not walk alone.** *BMC Biol* 2008, **6**:27.

4. Weeks AR, Velten R, Stouthamer R: **Incidence of a new sex-ratio-distorting endosymbiotic bacterium among arthropods.** *Proc R Soc London B Biol Sci* 2003, **270**:1857–1865.

5. Regnery RL, Spruill CL, Plikaytis BD: **Genotypic identification of rickettsiae and estimation of intraspecies sequence divergence for portions of two rickettsial genes.** *J Bacteriol* 1991, **173**:1576–1589.

6. Von der Schulenburg JHG, Habig M, Sloggett JJ, Webberley KM, Bertrand D, Hurst GDD, Majerus MEN: **Incidence of male-killing *Rickettsia* spp. (alpha-Proteobacteria) in the Ten-Spot Ladybird Beetle *Adalia decempunctata* L . (Coleoptera: Coccinellidae)**. *Appl Environ Microbiol* 2001, **67**:270–277.

7. Montenegro H, Solferini VN, Klaczko LB, Hurst GDD: **Male-killing *Spiroplasma* naturally infecting *Drosophila melanogaster*.** *Insect Mol Biol* 2005, **14**:281–287.

8. Baldo L, Dunning Hotopp JC, Jolley KA, Bordenstein SR, Biber SA, Choudhury RR, Hayashi C, Maiden MCJ, Tettelin H, Werren JH: **Multilocus sequence typing system for the endosymbiont *Wolbachia pipientis*.** *Appl Environ Microbiol* 2006, **72**:7098–7110.
